# Supplementary material for: Tuning SERS Signal via Substrate Structuring: Valves of Different Diatom Species with Ultrathin Gold Coating
Source: Nanomaterials (Basel). 2023 May 10;13(10):1594. doi: 10.3390/nano13101594 (PMC10221721; doi:10.3390/nano13101594)
Supplement: Supplementary file 1 [file nanomaterials-13-01594-s001.zip › nanomaterials-2378439-supplementary.pdf]

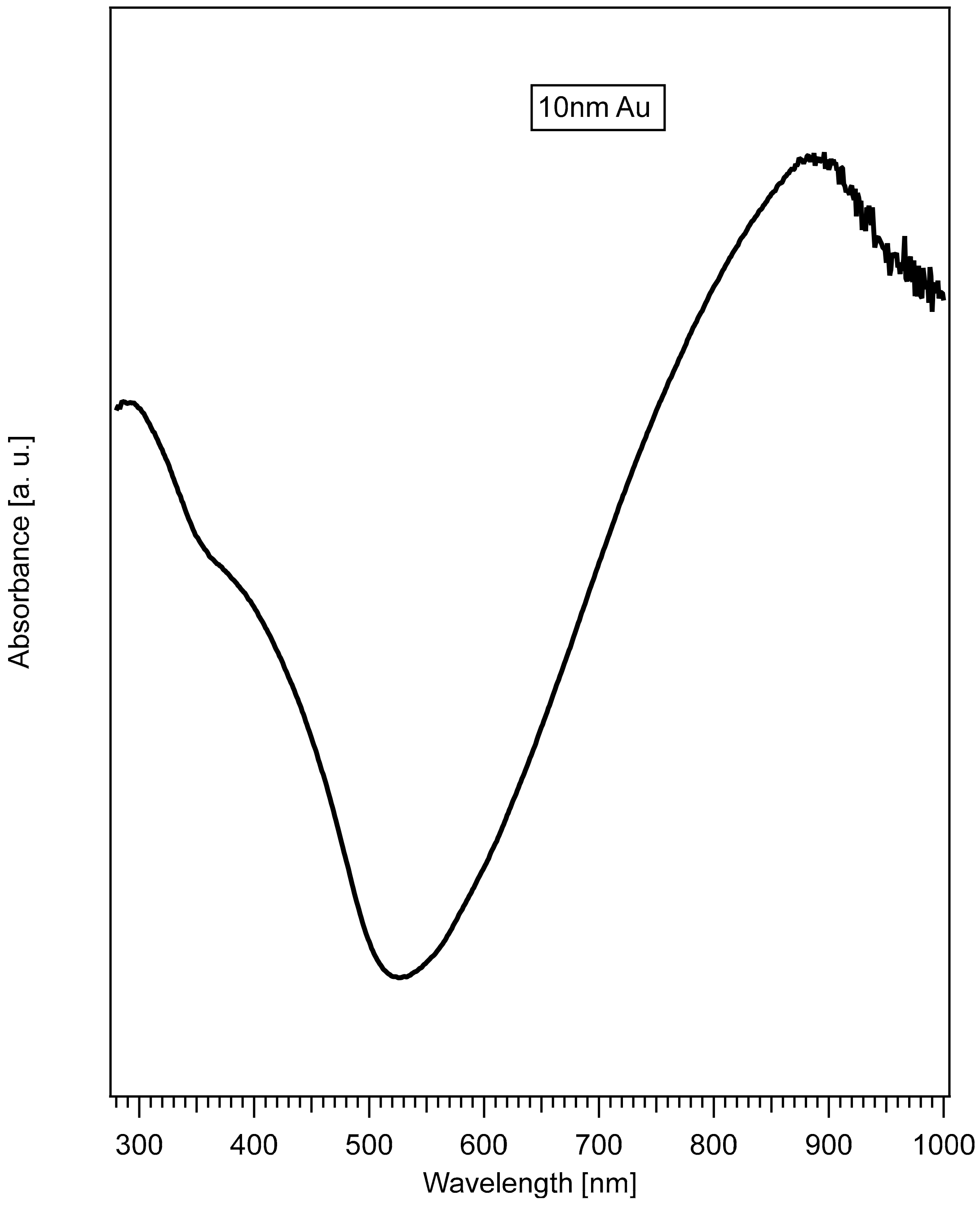

Figure S1: UV – VIS Absorbance spectrum of a 10 nm gold film on a glass slide activated with MPTMS.

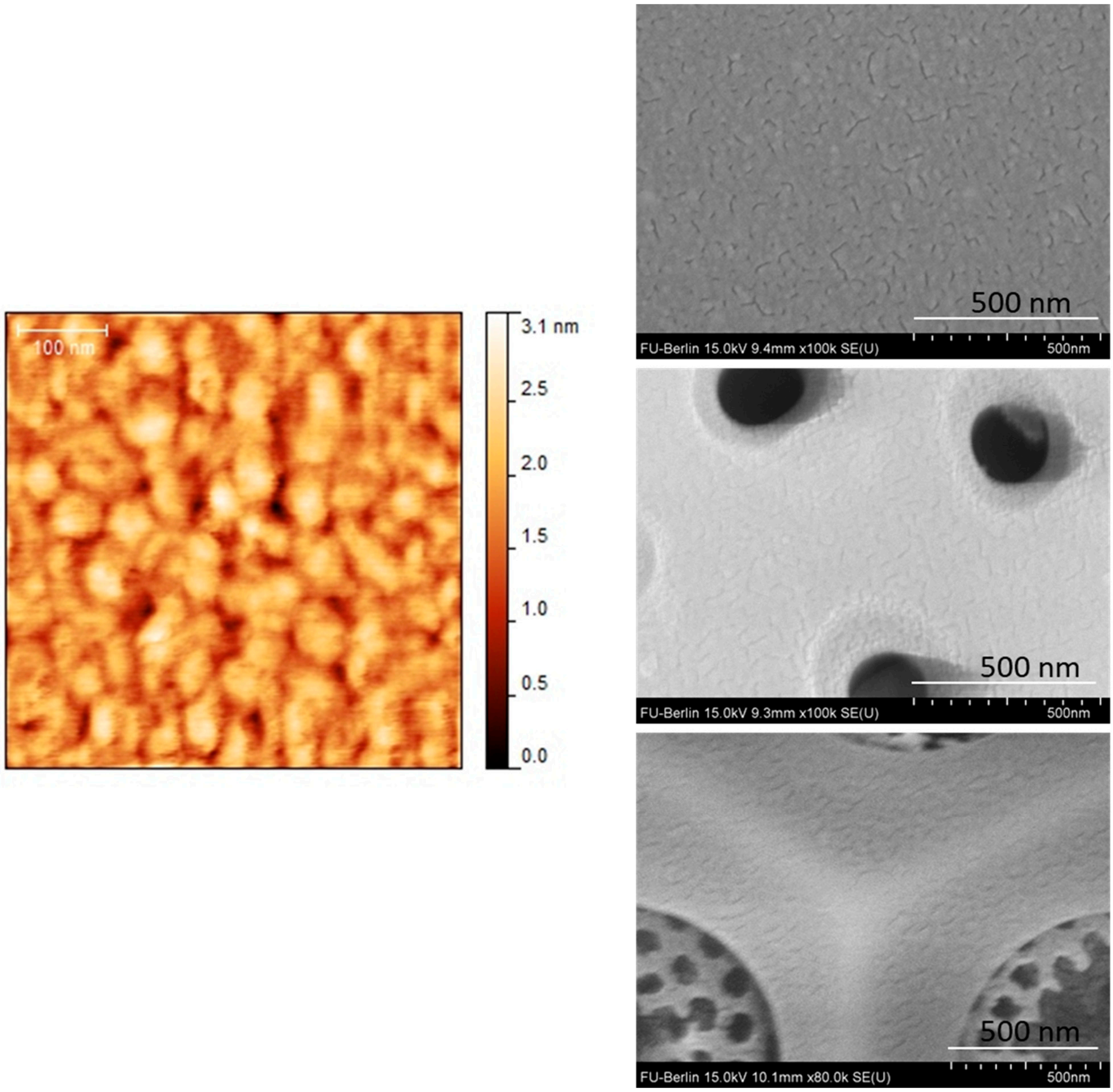

Figure S2: AFM image of the 10 nm gold film on a glass slide (left); and SEM micrographs of the gold film on a glass slide (top right), Aula valve (middle right) and Cosc valve (bottom right). A glass slide was activated with MPTMS before the gold layer was evaporated.

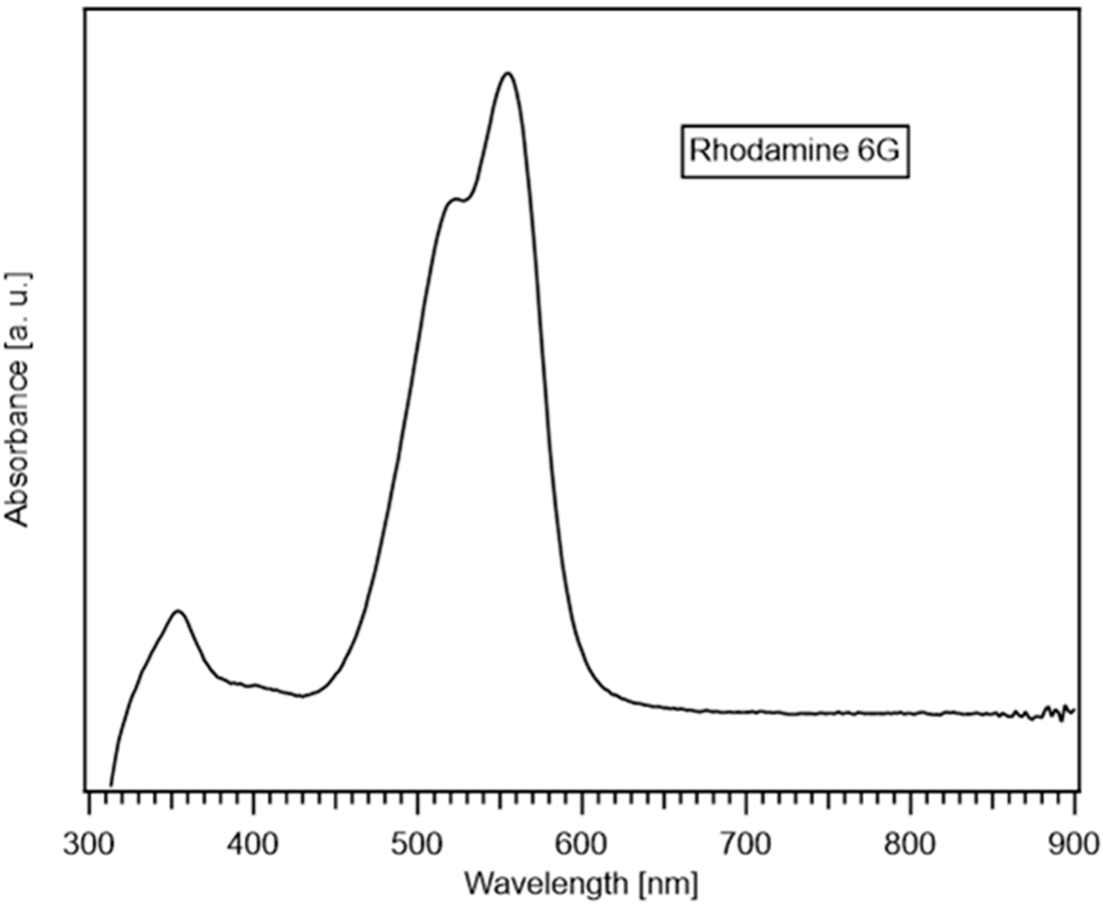

Figure S3: UV – VIS Absorbance spectrum of 10^{-3} M Rhodamine 6G dissolved in ethanol.

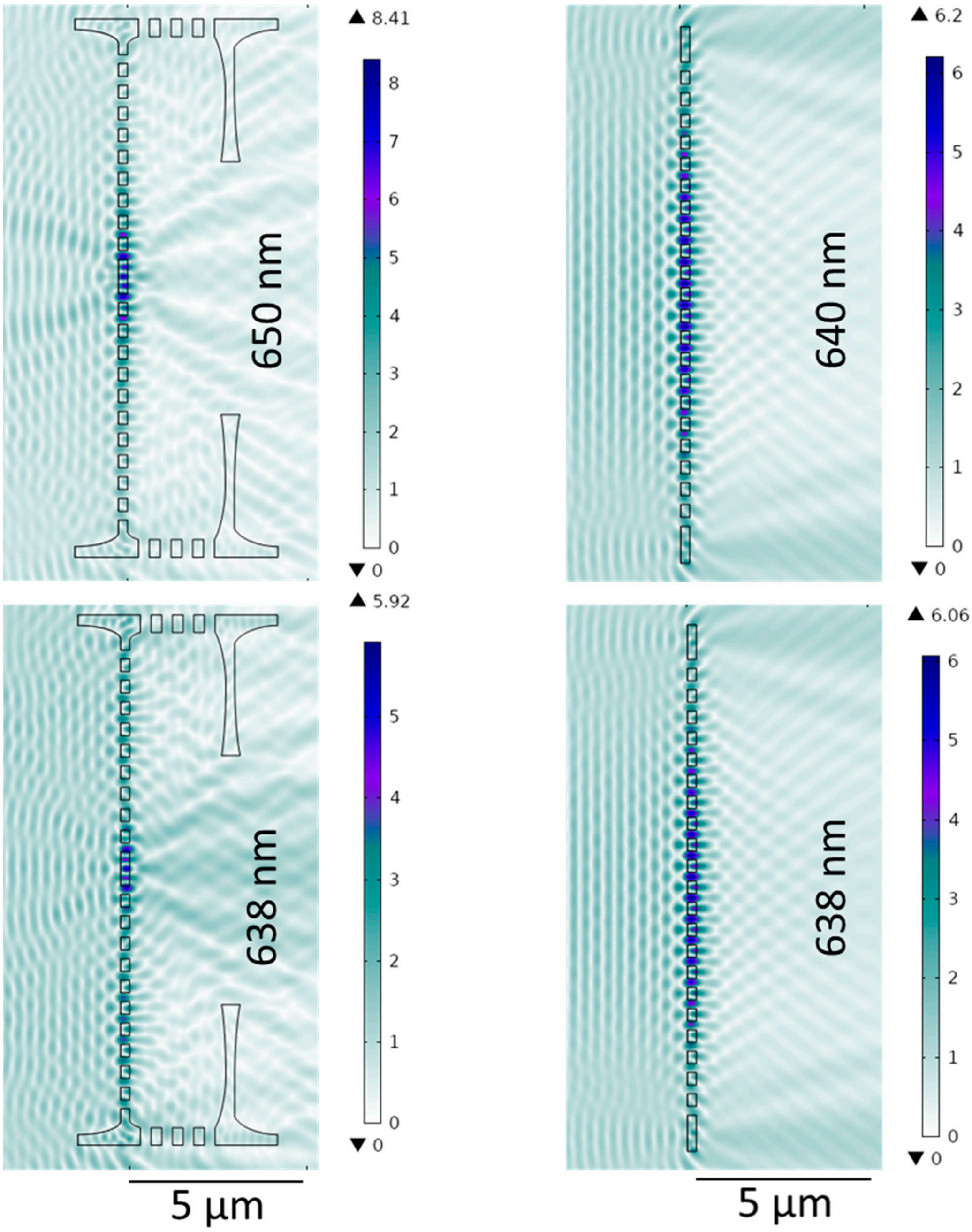

Figure S4: The influence of defects (left) and the absence of curved edges (right) on GMR in Aula valve.
